# The outcome of watchful waiting in patients with previously treated follicular lymphoma

**DOI:** 10.1002/cam4.4588

**Published:** 2022-02-07

**Authors:** Takahiro Fujino, Dai Maruyama, Akiko‐Miyagi Maeshima, Yo Saito, Hanae Ida, Rika Hosoba, Sayako Yuda, Shinichi Makita, Suguru Fukuhara, Wataru Munakata, Tatsuya Suzuki, Junya Kuroda, Koji Izutsu

**Affiliations:** ^1^ Department of Hematology National Cancer Center Hospital Tokyo Japan; ^2^ Division of Hematology and Oncology Kyoto Prefectural University of Medicine Kyoto Japan; ^3^ Department of Pathology National Cancer Center Hospital Tokyo Japan

**Keywords:** lymphoma, follicular, lymphoma, non‐Hodgkin, neoplasm regression, spontaneous, rituximab, watchful waiting

## Abstract

Watchful waiting (WW) is one of the standard approaches for newly diagnosed follicular lymphoma (FL) patients with low‐tumor burden. However, the impact of WW in FL patients at the first progression, remains unclear. We reviewed 206 FL patients who experienced the first progression after responding to the initial treatment at our institution between 1998 and 2017. Patients were classified into either the WW cohort (132 patients) or the immediate treatment cohort (74 patients). Overall, the median follow‐up from the first progression was 79.8 months (range, 2.1–227.0 months). In the WW cohort, the estimated median time to next treatment (TNT) was 19.7 months (95% confidence interval [CI], 13.4–30.2), and 76.5% (95% CI, 68.0–84.1) of the patients subsequently underwent the second‐line treatment at 5 years. There was a significant difference in the median time to treatment failure in the WW cohort (72.8 months; 95% CI, 64.6–94.0) compared to the immediate treatment cohort (23.3 months; 95% CI, 13.4–38.8) (HR, 2.13; 95% CI, 1.48–3.06), whereas overall survival and the cumulative incidence of histological transformation were not significantly different between two cohorts. In a multivariate analysis, rituximab refractory status, progression of disease within 24 months from the induction of first‐line therapy, and a high Follicular Lymphoma International Prognostic Index score at diagnosis were significantly associated with shorter TNT. Interestingly, 15 patients (11%) of the WW cohort experienced spontaneous tumor regression during WW, and their TNT (median, 82.1 months, 95% CI, 11.7‐NA) was longer than that of the remaining patients in the WW cohort (median, 16.5 months, 95% CI, 13.0–25.4), with a significant difference (*p* = 0.01). The results of the present study suggested that WW could be a safe and reasonable option even at the first progression for the selected FL patients, without a negative impact on clinical outcomes.

## Graphical abstract

We evaluated the impact of watchful waiting (WW) for patients with Follicular lymphoma who were at the first progression. Our study suggested that WW is feasible and safely postpones subsequent treatment for selected patients, even at the first progression. Also, WW has no negative impact on time to treatment failure, overall survival, and histological transformation risk.

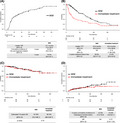

## 1 INTRODUCTION

Follicular lymphoma (FL) is one of the most common indolent lymphoma subtypes.^1^, ^2^ The survival outcome of FL has improved since the implementation of anti‐CD20 antibodies, such as rituximab, and the median overall survival (OS) has nearly reached 20 years.^3^ However, even when treated with rituximab‐containing chemotherapy, FL is incurable and would subsequently progress or relapse. The prognosis after relapse varies among patients because of the biological heterogeneity of FL. However, some patients become resistant to subsequent chemotherapy, and progression‐free survival (PFS) and OS tend to shorten with the repeated disease relapse.^4^ Presently, lymphoma itself is still the most frequent cause of death, especially for patients who experience transformation into aggressive lymphomas.^5^, ^6^ Also, the increase of adverse events due to repeated chemotherapy, including infection or the second primary malignancy, is concerning.^5^, ^7^

Watchful waiting (WW) has been traditionally conducted for asymptomatic FL patients^8^, ^9^, ^10^, ^11^, ^12^ and is now widely accepted as a standard approach for newly diagnosed FL patients with low‐tumor burden.^13^ WW has also been applied to FL patients who experienced the first progression in daily practice, predominantly when asymptomatic. However, the rationale for applying WW for relapsed FL patients is attributed to an empirical basis, and clinical outcomes of patients who underwent WW at the first progression have rarely been reported.

To elucidate the clinical value of WW for patients with FL at the first relapse, we investigated the clinical features of patients with relapsed FL who were subjected to WW at the first progression, and evaluated the impact of WW on subsequent clinical outcomes.

## 2 SUBJECTS AND METHODS

### 2.1 Study design

**FIGURE 1:**
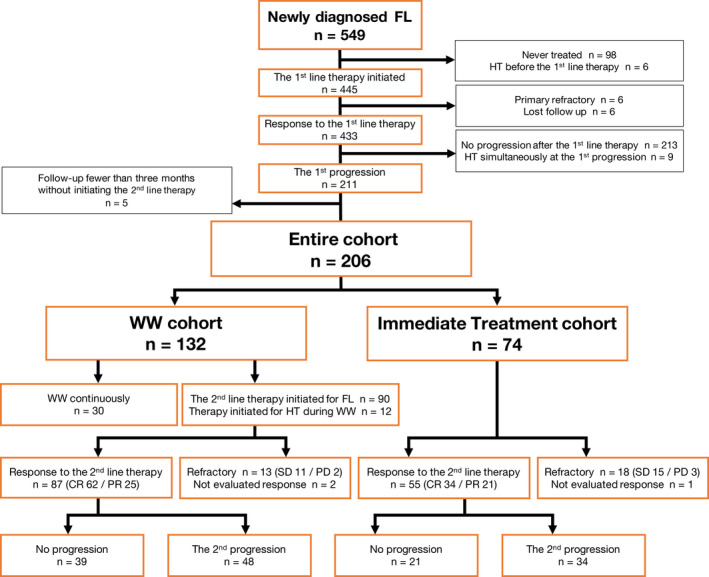
Patient selection flow chart. CR, complete response; FL, follicular lymphoma; HT, histological transformation; PD, progressive disease; PR, partial response; SD, stable disease; WW, watchful waiting

We reviewed the pathological database at the National Cancer Center Hospital and extracted 549 patients with newly diagnosed FL grade 1–3A at our institution between January 1998 and December 2017. We retrospectively reviewed each patient's chart, selected 206 patients whose disease had progressed after responding to the first‐line therapy, and determined the date of the first progression. Regarding the imaging study at our institution, patients who responded to the first‐line therapy have regularly had routine surveillance with computed tomography (CT) every 6 months for 5 years until progression, so we used radiologists' reports to reconfirm the date of disease progression according to the International Working Group Guidelines of 2007.^14^ The following criteria were used for exclusion of patients; never treated since initial diagnosis (*n* = 98), confirmed histological transformation before initiating the first‐line therapy (*n* = 6) or simultaneously at the first progression (*n* = 9), primary refractory to the first‐line therapy (*n* = 6), without disease progression after the first‐line therapy (*n* = 213), follow‐up fewer than 3 months after the first progression without initiating the second‐line treatment (*n* = 5), or lost follow up (*n* = 6) (Figure 1).

Then, we checked whether the treatment‐free follow‐up had been conducted longer than 3 months or a second‐line therapy had been initiated immediately. We defined the WW cohort as patients who remained treatment‐free at least 3 months after the first progression, while the immediate treatment cohort as patients who initiated the second‐line treatment within 3 months of the detection of the first progression, in accordance with the criteria used for the newly diagnosed FL patients in the previous studies.^10^, ^12^, ^15^

Also, we defined the high tumor burden (HTB) according to the Groupe d'Etude des Lymphomes Folliculaires criteria,^9^ including elevated lactate dehydrogenase (LDH). Rituximab refractory status was defined as progression during any rituximab‐containing regimen or within 6 months from the last rituximab dose in the induction or maintenance settings. Progression of disease within 24 months from the initiation of the first‐line therapy was defined as POD24.

### 2.2 Statistical analysis

We analyzed the following endpoints: time to next treatment (TNT), time to treatment failure (TTF), OS, and the risk of histological transformation. TNT was defined as the time from the first progression to the initiation of second‐line treatment, and was illustrated by the cumulative incidence method and estimated by Gray's test only in the WW cohort, using death as a competing risk. TTF was defined as the time from the first progression to the following events, whichever comes first; disease progression after the second‐line treatment, the initiation of the third‐line treatment, or death by any cause, and calculated for both cohorts (Figure S1). OS was defined as the time from the first progression, to death by any cause. TTF and OS were estimated using the Kaplan–Meier method and were compared with the log‐rank test. The risk of histological transformation from the first progression was assessed using the cumulative incidence method and was compared with Gray's test, using death as a competing risk. Besides, the relevance between clinical factors and TNT was analyzed using the Fine‐Gray proportional hazards models in the WW cohort. The results were shown as the hazard ratio (HR) and 95% confidence interval (CI), and *p*‐value. Two‐sided *p* < 0.05 were considered statistically significant. All analyses were performed with EZR version 1.33 (Saitama Medical Center, Jichi Medical University), a graphical user interface for R (The R Foundation for Statistical Computing).^16^ The attending physicians conducted all the decision‐making. This study was approved by the Institutional Review Board of the National Cancer Center and was conducted in accordance with the principles of the Declaration of Helsinki.

## 3 RESULTS

### 3.1 Patient characteristics at the initial diagnosis

Of 206 patients included in this study, 132 patients (64%) were categorized into the WW cohort and 74 patients (36%) into the immediate treatment cohort. The median follow‐up time of all 206 patients was 134.8 months (range, 23.1–266.8 months) from the initial diagnosis and 79.8 months (range, 2.1–227.0 months) from the first progression.

Patient characteristics are shown in Table 1. Overall, 94 patients (46%) were male. At the initial diagnosis, the patients' median age in the WW cohort and that of the immediate treatment cohort were 58 and 57 years old, respectively. In each cohort, 63% of patients had HTB. Ninety‐seven patients (73%) in the WW cohort were in an advanced clinical stage, while 63 patients (85%) were in the immediate treatment cohort.

**TABLE 1:** Patient characteristics at the initial diagnosis and the first progression, including first and second‐line therapy information

		Entire cohort (*n* = 206)	WW (*n* = 132)	Immediate treatment (*n* = 74)	*p*‐value
Status at the initial diagnosis					
Gender	Male/female, *n* (%)	94 (46)/112 (54)	61 (46)/71 (54)	33 (45)/41 (55)	0.88
Age	Median (range)	58 (19–85)	58 (19–85)	57 (34–76)	0.97
Year of initial diagnosis	Median (range)	2006 (1998–2017)	2006 (1998–2017)	2007 (1998–2017)	0.84
Tumor burden^a^	High, *n* (%)	130 (63)	83 (63)	47 (63)	1.00
Clinical stage	Advanced, *n* (%)	160 (78)	97 (73)	63 (85)	0.06
Bone marrow involvement	Yes, *n* (%)	98 (48)	61 (46)	37 (50)	0.66
FL grade	3A, *n* (%)	40 (19)	23 (17)	17 (23)	0.36
FLIPI score	High, *n* (%)	55 (27)	33 (25)	22 (30)	0.51
The 1st‐line therapy					
WW before the 1st‐line therapy	Yes, *n* (%)	56 (27)	35 (27)	21 (28)	0.87
	Duration (month); median (range)	9.4 (3.1–111.2)	6.7 (3.1–71.4)	13.9 (4.8–111.2)	**0.02**
Therapy	Chemotherapy ± Rituximab, *n* (%)	150 (73)	91 (69)	59 (80)	
	Rituximab alone, *n* (%)	29 (14)	21 (16)	8 (11)	0.46
	Radiation alone, *n* (%)	22 (11)	16 (12)	6 (8)	
	Others, *n* (%)	5 (2)	4 (3)	1 (1)	
Response to the 1st‐line therapy	CR, *n* (%)	154 (75)	105 (80)	49 (66)	**0.05**
Initiation of ritrximab maintenance	Yes, *n* (%)	20 (9)	9 (7)	11 (15)	**0.05**
Status at the 1st progression					
Age	Median (range)	62 (26–94)	63 (26–94)	62 (37–81)	0.70
ECOG‐PS	0/1/2, *n* (%)	188 (91)/17 (8)/1 (1)	125 (95)/7 (5)/0 (0)	63 (85)/10 (14)/1 (1)	**0.03**
Cause of progression					
Increase by >50% of previously involved sites from nadir	Yes, *n* (%)	145 (70)	90 (68)	55 (74)	0.43
Any new lesion	Yes, *n* (%)	90 (44)	56 (42)	34 (46)	0.66
Tumor burden^a^	High, *n* (%)	71 (35)	35 (27)	36 (49)	**<0.01**
Elevated LDH	Yes, *n* (%)	43 (21)	24 (18)	19 (26)	0.22
Re‐biopsy at the 1st progression	Yes, *n* (%)	73 (35)	50 (38)	23 (31)	0.37
Rituximab‐naive	Yes, *n* (%)	31 (15)	20 (15)	11 (15)	1.00
Rituximab refractory^b^	Yes, *n* (%)	19 (9)	7 (5)	12 (16)	**0.01**
POD24	Yes, *n* (%)	77 (37)	39 (30)	38 (51)	**<0.01**
The 2nd‐line therapy					
Spontaneous regression before the 2nd‐line therapy	Yes, *n* (%)	15 (7)	15 (11)	NA	NA
Therapy	Rituximab alone, *n* (%)	43 (21)	24 (18)	19 (26)	
	Bendamustine ± Rituximab, *n* (%)	29 (14)	22 (17)	7 (10)	
	CHOP ± Rituximab, *n* (%)	19 (9)	14 (11)	5 (7)	
	Fludarabine ± Rituximab, *n* (%)	15 (7)	3 (2)	12 (16)	NA
	Ibritumomab tiuxetan, *n* (%)	12 (6)	8 (6)	4 (5)	
	Others, *n* (%)	58 (28)	31 (23)	27 (36)	
	Continuous WW, *n* (%)	30 (15)	30 (23)	NA	
Consolidative transplantaion	Autologous, *n* (%)	4 (2)	2 (2)	2 (3)	0.67
	Allogenic, *n* (%)	2 (1)	1 (1)	1 (1)	

Bold values indicate statistical significance of *p*‐value.

Abbreviations: CR, complete response; ECOG‐PS, Eastern Cooperative Oncology Group Performance Status Scale; FL, follicular lymphoma; FLIPI, Follicular Lymphoma International Prognostic Index; LDH, lactate dehydrogenase; POD24, progression of disease within 24 months from the initiation of the first‐line therapy; WW, watchful waiting.

^a^ Judged by the Groupe d'Etude des Lymphomes Folliculaires (GELF) criteria, including elevated LDH.

^b^ Progression during any rituximab‐containing regimen or within 6 months of the last rituximab dose in the induction or maintenance setting.

### 3.2 The first‐line therapy

Before initiating the first‐line treatment, 56 patients (27%) were subjected to the first‐line WW, and the median duration of the first‐line WW was 9.4 months (range, 3.1–111.2 months). Regarding the first‐line therapy, 150 (73%), 29 (14%), and 22 patients (11%) underwent chemotherapy with or without rituximab, rituximab monotherapy, or radiation alone, respectively. The proportion of patients who underwent rituximab maintenance therapy following the first‐line therapy in the WW cohort and the immediate treatment cohort were 7% and 15%, respectively. As a result, patients who achieved a complete response (CR) to the first‐line therapy in the WW cohort and the immediate treatment cohort were 80% and 66%, respectively.

### 3.3 Patient status at the first progression

The patient characteristics at the first progression are also shown in Table 1. The median age of all patients at the first progression was 62 years old (range, 26–94 years old). All but one patient had Eastern Cooperative Oncology Group (ECOG) performance status better than one. There was a trend of more favorable performance status in the WW cohort compared with the immediate treatment cohort. At the first progression, 70% of all patients experienced a ≥50% increase of tumor size at the previously involved site, and 44% showed the emergence of a new lesion. Patients with HTB at the first progression in the WW cohort and the immediate treatment cohort were 27% and 49%, and those with elevated LDH were 18% and 26%, respectively. Re‐biopsy to exclude the histological transformation was conducted in 35% of all patients. Rituximab‐naive patients at the first progression were 15% of patients in both cohorts. Seven patients (5%) in the WW treatment cohort, and 12 patients (16%) in the immediate treatment cohort, fulfilled the criteria of rituximab refractory at the first progression. The median duration from the first‐line therapy to the first progression in the WW cohort and the immediate treatment cohort was 36.3 and 23.7 months, respectively (Table S1). POD24 in the WW cohort and the immediate treatment cohort was 30% and 51%, respectively.

### 3.4 WW at the first progression

**FIGURE 2:**
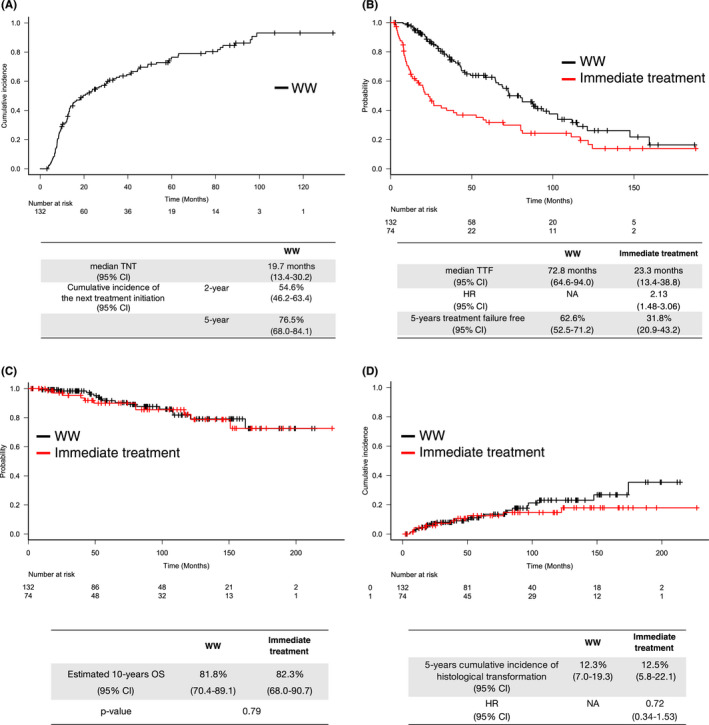
(A) Time to next treatment for the WW cohort. (B) Time to treatment failure for the both cohorts. (C) Overall survival for the both cohorts. (D) Cumulative incidence of pathological transformation, using death as competing risk for the both cohorts. CI, confidence interval; HR, hazard ratio; NA, not available; OS, overall response; TNT, time to next treatment; TTF, time to treatment failure; WW, watchful waiting

Of 132 patients in the WW cohort, the median duration of WW at the data cut‐off was 17.3 months (range, 3.1–133.5 months), and the estimated median TNT was 19.7 months (95% CI, 13.4–30.2). Subsequently, 102 patients (77%) had received second‐line treatment by the data cut‐off. The proportion of patients who underwent the second‐line treatment at 6 months, 1 year, 2 years, and 5 years was estimated to be 7.6% (95% CI, 4.2–13.7), 32.1% (95% CI, 24.8–40.9), 54.6% (95% CI, 46.2–63.4) and 76.5% (95% CI, 68.0–84.1), respectively (Figure 2A). Notably, 30 patients (23%) in the WW cohort continued WW at the data cut‐off with a median duration of 39.7 months (range, 3.1–133.5 months).

### 3.5 Clinical outcomes

There was a significant difference in the estimated median TTF in the WW cohort (72.8 months; 95% CI, 64.6–94.0) and the immediate treatment cohort (23.3 months; 95% CI, 13.4–38.8) (HR, 2.13; 95% CI, 1.48–3.06). On the other hand, the estimated 10‐year OS in the WW cohort and the immediate treatment cohort was 81.8% (95% CI, 70.4–89.1) and 82.3% (95% CI, 68.0–90.7), respectively (*p* = 0.79) (Figure 2B,C).

The cumulative incidence of histological transformation was estimated to be 12.3% (95% CI, 7.0–19.3) in the WW cohort and 12.5% (95% CI, 5.8–22.1) in the immediate treatment cohort at 5 years from the first progression (HR, 0.72; 95% CI, 0.34–1.53) (Figure 2D). In the WW cohort, 12 patients (52% of all events of histological transformation in this cohort) were confirmed histological transformation just during WW. Among these 12 patients, the median duration of WW was 19.5 months (range, 7.9–96.6) from the first progression. Also, the evaluation by fluorodeoxyglucose‐positron emission tomography‐computed tomography (FDG‐PET/CT) at the first progression, was conducted for nine patients (75%), and the standard uptake value max (SUVmax) ranged from 1.98 to 14.7 (median, 6.6). In addition, a re‐biopsy to exclude histological transformation was done for seven patients (56%). In the end, 10 of 12 patients (83%) underwent either FDG‐PET/CT or re‐biopsy at the first progression.

### 3.6 The second‐line therapy

Rituximab monotherapy was most frequently conducted in both cohorts as a second‐line treatment, with 18% in the WW cohort and 26% in the immediate treatment cohort. Following the second‐line treatment, consolidation hematopoietic stem cell transplantation was conducted in six patients (3%), including four patients with autologous and two patients with allogeneic transplantation. Of these six patients, histological transformation was confirmed in three, whereas the other three patients were diagnosed as clinically transformed FL.

### 3.7 Prognostic factors for TNT

**FIGURE 3:**
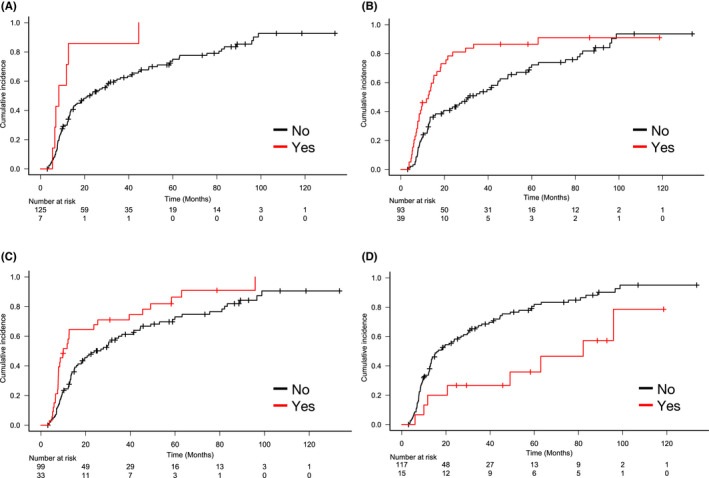
Time to next treatment for the WW cohort, stratified by each prognostic factor. (A) Rituximab refractory (B) POD24 (C) The high score of FLIPI at the initial diagnosis (D) Spontaneous regression during WW. FLIPI, Follicular Lymphoma International Prognostic Index; POD24, progression of disease within 24 months from the initiation of the first‐line therapy; WW, watchful waiting

To explore the prognostic factors for TNT, we conducted a univariate analysis based on patients' characteristics and clinical factors. Rituximab refractory status, POD24, and a high score (≥3) on the Follicular Lymphoma International Prognostic Index (FLIPI) at the initial diagnosis were found to be significantly associated with a shorter TNT. Multivariate analysis using significant variables (*p* < 0.1 in univariable analysis), revealed that rituximab refractory status, POD24, and a high FLIPI score at the initial diagnosis, had a significant impact on TNT, with HR 2.60 (95% CI 1.33–5.06), HR 1.72 (95% CI 1.04–2.83), and HR 1.88 (95% CI 1.19–2.98), respectively (Table. 2; Figure 3A–C). Notably, these three variables were preserved as significant prognostic factors when rituximab‐naive 20 patients (15%) at the first progression were excluded (data not shown).

**TABLE 2:** Univariate and multivariate analysis for prognostic factors for time to next treatment (TNT)

	Univariate			Multivariate		
	HR	95% CI	*p*‐value	HR	95% CI	*p*‐value
Status at the initial diagnosis						
High tumor burden	1.13	0.75–1.71	0.57		NA	
Advanced clinical stage	1.08	0.67–1.75	0.75		NA	
Bone marrow involvement	0.85	0.58–1.26	0.42		NA	
FL grade 3A	1.10	0.67–1.82)	0.71		NA	
FLIPI score: High (3–5)	2.00	1.27–3.13	**< 0.01**	1.88	1.19–2.98	**< 0.01**
The 1st‐line therapy						
Not R‐chemotherapy	0.87	0.56–1.35	0.53		NA	
Non CR to the 1st line therapy	1.43	0.94–2.17	0.096	0.98	0.59–1.61	0.93
Status at the 1st progression						
>60 years old	1.27	0.86–1.88	0.23		NA	
High tumor burden	1.20	0.81–1.77	0.37		NA	
Elevated LDH	1.12	0.70–1.79)	0.64		NA	
Relapse of the known lesion	1.06	0.68–1.63	0.81		NA	
Emerging new lesion	1.02	0.69–1.51	0.91		NA	
Rituximab refractory	2.94	1.36–6.34	**< 0.01**	2.60	1.33–5.06	**< 0.01**
POD24	1.77	1.12–2.80	**0.015**	1.72	1.04–2.83	**0.03**

*Note*: Variables with significance *p* < 0.1 in univariable analysis were included in the multivariable analyses. Bold values indicate statistical significance of *p*‐value.

Abbreviations: 95% CI, 95% confidence interval; CR, complete response; FL, follicular lymphoma; FLIPI, Follicular Lymphoma International Prognostic Index; HR, hazard ratio; LDH, lactate Dehydrogenase; POD24, progression of disease within 24 months from the initiation of the first‐line therapy.

### 3.8 Cause of death

Death occurred in 16 patients (12%) in the WW cohort and 11 patients (15%) in the immediate treatment cohort. In each cohort, lymphoma was the leading cause of death (eight patients [50%] in the WW cohort and six patients [55%] in the immediate treatment cohort), followed by the second primary malignancy (six patients (38%) in the WW cohort and two patients (18%) in the immediate treatment cohort; Figure S2). Of eight patients who died from lymphoma in the WW cohort, histological transformation was confirmed in six (75%); of which, two patients were confirmed just during WW at 80.9 and 96.6 months each, whereas the remaining four patients were confirmed after the initiation of the second‐line treatment, with a median duration from the first progression of 57.0 months (range, 50.3–105.6 months). All eight patients who died from lymphoma in the WW cohort had undergone a second‐line treatment, but the overall response rate was only 50%. The median OS of these eight patients from the first progression was 70.6 months (range, 45.8–121.1 months).

### 3.9 Spontaneous tumor regression

Interestingly, 15 patients (11%) of the WW cohort experienced spontaneous tumor regression (partial or CR) during WW. TNT of patients who experienced spontaneous tumor regression (median, 82.1 months, 95% CI, 11.7‐NA) was longer than that of the remaining patients in the WW cohort (median, 16.5 months, 95% CI, 13.0–25.4), with a significant difference (*p* = 0.01, Figure 3D). The evaluation by FDG‐PET/CT at the first progression was conducted for 10 patients (67%), and the median SUVmax was 6.9 (range, 2.8–14.2). Notably, 14 patients (93%) were confirmed the first progression in the state of low‐tumor burden. Also, no patients with spontaneous tumor regression died, with a median follow‐up of 88.5 months (range 21.0–189.3) from the first progression.

## 4 DISCUSSION

WW has been recognized as a standard of care for newly diagnosed patients with advanced‐stage, low‐tumor burden FL.^8^, ^9^, ^10^, ^11^, ^12^, ^13^ However, the impact of WW at the first progression on subsequent clinical outcomes has not been elucidated in relapsed FL patients with prior treatment history.

In this single‐institution study, we found that WW could be a clinical option, even at the first progression, for patients with relapsed FL, without having a negative impact on clinical outcomes. In our study, the median TNT in the WW cohort was as long as 19.7 months, and WW at the first progression for the selected patients did not negatively affect TTF, OS, or the cumulative incidence of histological transformation. In addition, rituximab refractory status, POD24, and a FLIPI score of 3–5 at the initial diagnosis, were significantly associated with shorter TNT.

The impact of WW on histological transformation is of great interest. Regarding first‐line treatment, the answer to this question is still controversial. Using the pooled data from prospective clinical trials and population‐based registries, the Aristotle study reported that the risk of histological transformation was significantly higher in the WW cohort and was significantly reduced by immediate induction of rituximab.^15^ However, a randomized phase 3 trial conversely showed that WW for advanced‐stage, low tumor burden FL patients did not significantly increase the frequency of histological transformation compared to the induction of rituximab monotherapy.^17^ Notably, these two studies both targeted newly diagnosed FL patients. To our knowledge, our current study is the first report investigating the impact of WW on the emergence of histological transformation following the first progression, in FL patients. It is true that 52% of all cases of histological transformation in the WW cohort occurred just during WW. However, the cumulative incidence of histological transformation was not statistically significant between the two cohorts, therefore we could conclude that WW for selected patients at the first progression does not negatively affect the risk of histological transformation. Nevertheless, considering that the median duration from the first progression to the confirmation of histological transformation during WW was 19.5 months (range, 7.9–96.6), careful observation of clinical behavior is needed throughout the clinical course.

We extracted three prognostic factors associated with shorter TNT in the WW cohort: a high FLIPI score at the initial diagnosis, POD24, and rituximab refractory status. In recent years, POD24 has been regarded as a solid prognostic factor for early mortality of FL patients.^18^ Since this prognostic factor has been prevalent in the field of routine medical care, there is a possibility that the attending physician tended to introduce the second‐line therapy faster for patients who fulfilled the criteria of POD24, and as a result, TNT of these patients might become shorter. In this study, TNT of patients who fulfilled POD24 was significantly shorter than those who did not. However, the median TNT of patients who fulfilled POD24 was 12.6 months (range, 3.8–118.5), which seems longer than expected. This is possibly because our patients were so heterogeneous, for example, a variety of first‐line therapy including the first‐line WW or various tumor burden, that the magnitude of POD24 might become weakened. However, considering that POD24 is established as the prognostic factor for OS of FL patients, we think it seems reasonable that POD24 was extracted as a significant factor for shorter TNT. Compared to POD24, the prognostic impact of rituximab refractory status has yet to be established. However, a study of bendamustine monotherapy reported that the previous refractoriness to the rituximab‐containing regimen was associated with a shorter PFS of relapsed FL.^19^, ^20^ Together, the clarification of how rituximab resistance impairs the prognosis of FL is an important future area of research.

It was impressive that 30% of patients in the WW cohort had fulfilled POD24 at the first progression. First, as a premise, we have modified the definition of POD24 in this study to be disease progression within 24 months “from the initiation of the first‐line therapy” rather than “from the initial diagnosis” as the previous study defined in their original study, because our study includes patients who underwent the first‐line WW, whereas the previous study excluded them.^18^ In our research, among 39 patients who underwent WW at the first progression while meeting POD24, the proportion of patients with HTB was as low as 18% (data not shown), whereas that in the immediate treatment cohort were 49%. Our institution has performed surveillance CT on FL patients after initial treatment, which might lead more patients to be detected their first progression of disease meeting POD24 with a low tumor burden. Importantly, although POD24 is indeed an important prognostic factor of OS in FL, it is not an absolute criterion for the intervention of the second‐line therapy. As a result, we have shown that WW is feasible at the first progression for some patients, even fulfilling POD24 if we consider their tumor burden or the indolent clinical course after detection of the first progression. Furthermore, Japanese medical insurance had not supported rituximab maintenance therapy for FL until 2015, whereas it had already become the standard of care in Western countries much earlier, which may have affected PFS and contributed to the large number of patients who met POD24 in our study.

In our study, 15 patients (11%) in the WW cohort achieved spontaneous tumor regression during WW. It was once reported that spontaneous tumor regression occurred in 18 of 140 patients (12.8%) of FL, including six (4%) of CR patients.^21^ It was also reported that during WW at the initial diagnosis for advanced‐stage, low tumor burden FL patients, spontaneous tumor regression occurred in 12% of patients, including 6% of CR patients.^17^ However, these previous studies were conducted for patients with newly diagnosed FL and the subsequent clinical outcomes of these patients were not reported in detail. Herein, we showed that the clinical outcomes of patients who experienced spontaneous tumor regression during WW following the first progression was excellent, with significantly longer TNT (median, 62.9 months) than others in the WW cohort and no mortality during a median follow‐up of 88.5 months from the first progression. Strictly, we should consider the possibility of a differential diagnosis different from relapse of FL (i.e., non‐malignant condition), and the additional biopsy should have been conducted to determine that this phenomenon was truly the spontaneous regression. Nevertheless, we think that our result suggested a better prognosis for patients who might experience spontaneous tumor regression, even after the first progression.

Our study has several limitations, including the retrospective nature of the analysis. This study was a single‐institution analysis with a small number of patients. Thus, the number of patients may not be sufficiently powered to compare the clinical outcomes of patients in the WW cohort and the immediate treatment group. Also, our clinical practice of surveillance CT might be useful for identifying patients who relapsed in a state of low tumor burden, and as many as 64% of all analyzed patients might be sorted to the WW cohort.

## 5 CONCLUSION

Our study suggested that WW in patients with FL at the first progression is feasible and safely postpones subsequent treatment for selected patients. Moreover, WW has no negative impact on TTF, OS, and histological transformation risk. Further studies are needed to optimize the initiation of the second‐line treatment and find the optimal candidates for WW at the first progression.

## CONFLICT OF INTEREST

Dai Maruyama received research funding from Chugai Pharmaceutical and honoraria from Chugai Pharmaceutical and Zenyaku Kogyo. Wataru Munakata received research funding from Chugai Pharmaceutical. Junya Kuroda received a scholarship grant from Chugai pharmaceutical. Koji Izutsu received research funding from Chugai Pharmaceutical. The remaining authors declare no competing financial interests.

## AUTHOR CONTRIBUTIONS

Takahiro Fujino and Dai Maruyama designed the study. Takahiro Fujino, Dai Maruyama, Yo Saito, Hanae Ida, Rika Hosoba, Sayako Yuda, Shin‐ichi Makita, Suguru Fukuhara, Wataru Munakata, Tatsuya Suzuki, and Koji Izutsu provided the cases. Akiko‐Miyagi Maeshima reviewed the histopathological diagnosis. Takahiro Fujino performed the statistical analysis. Takahiro Fujino and Dai Maruyama wrote the draft of the manuscript. Junya Kuroda reviewed and revised the draft. All authors have read and approved the final version of the manuscript.

## ETHICS STATEMENT

The study design was approved by the appropriate ethics review board of the National Cancer Center, Tokyo, Japan. This study was conducted in accordance with the principles of the Declaration of Helsinki. We did not obtain formal consent from the patients because of the nature of the retrospective observational study. Instead, we provide all patients with the opportunity to opt‐out.

## Supporting information


Figure S1
Figure S2
Table S1

## Data Availability

The data that support the findings of this study are available on request from the corresponding author. The data are not publicly available due to privacy or ethical restrictions.
