# Supplementary material for: The outcome of watchful waiting in patients with previously treated follicular lymphoma
Source: Cancer Med. 2022 Feb 7;11(10):2106–16. doi: 10.1002/cam4.4588 (PMC9119349; doi:10.1002/cam4.4588)
Supplement: Supplementary file 1 — Figure S1 Figure S2 Table S1 [file CAM4-11-2106-s001.doc]

**Appendix:**

**Supplementary Figure 1.**

The definition of TNT and TTF. TNT is calculated for the WW cohort only.

TNT, time to next treatment; TTF, time to treatment failure; WW, watchful waiting

**Supplementary Figure 2.**

Cumulative incidence for the competing risks of cause of death for WW cohort and Immediate treatment cohort.

WW, watchful waiting; FL, follicular lymphoma; MDS, myelodysplastic syndromes; AML, acute myeloid leukemia

**Supplementary Table 1.**

Duration from the initiation of the first-line therapy to the confirmation of the first progression.

WW, watchful waiting
